# The national acceptance of pelvic congestion syndrome among other common venous pathologies

**DOI:** 10.1016/j.jvsv.2026.102596

**Published:** 2026-08-10

**Authors:** Aakanksha Gupta, Patrick D. Conroy, Chris Dehaven, Apurva Puli, Dejah Judelson, Joseph V. Lombardi, Laurel Hastings, Katherine McMackin

**Affiliations:** a Department of Vascular and Endovascular Surgery, Cooper University Hospital, Camden, NJ; b Division of Vascular and Endovascular Surgery, University of Massachusetts Medical Center, Worcester, MA; c Department of Vascular Surgery, AtlantiCare Regional Medical Center, Pomona, NJ; d Cooper Medical School of Rowan University, Camden, NJ

**Keywords:** PCS, Pelvic congestion syndrome, Ovarian vein embolization

## Abstract

**Background:**

Pelvic congestion syndrome (PCS) is characterized by chronic pelvic pain persisting for over 6 months that is exacerbated by sexual activity, menstruation, and prolonged standing. PCS is associated with ovarian and iliac varicose veins (VV) and primarily affects female patients of childbearing age, often resolving at menopause. The purpose of this study is to detect national trends of PCS, in comparison with other common venous pathologies, chronic venous insufficiency (CVI), and VV.

**Methods:**

Patient data were collected from the Healthcare Cost and Utilization Project's National Inpatient Sample 2004 to 2021, bridging International Classification of Diseases, Ninth Revision, Clinical Modification (ICD-9-CM) and International Classification of Diseases, 10th Revision, Clinical Modification (ICD-10-CM) codes for PCS (ICD-9, 625.5; ICD-10, N94.89), CVI (ICD9, 459.8; ICD-10, I87.2), VV (ICD-9, 454.x; ICD-10, I83.x), and pelvic varices (ICD-9, 456.5-6; ICD-10, I86.2-3), were isolated for analysis. Data extraction and statistical programming were conducted via Stata version 17.0 (StataCorp LLC).

**Results:**

During the years using the ICD-9 coding system, the prevalence of PCS decreased. However, with the transition to ICD-10, PCS prevalence began to rise, continuing to increase through 2021, from 8605 cases in 2016 to 9250 cases in 2021. Similarly, the prevalence of VV and CVI showed an increasing trend across the ICD-9 to ICD-10 transition period. VV cases rose from 44,185 in 2004 to 71,180 in 2021, and CVI cases increased from 173,430 to 232,850 over the same period. In contrast, the prevalence of pelvic varices decreased, from 2185 cases in 2004 to 990 cases in 2021. In addition, no major trends were observed in the prevalence of inpatient abdominal venous procedures or leg venous procedures, indicating a stable incidence rate for these conditions.

**Conclusions:**

This study highlights the rising prevalence of PCS, particularly the growth in national inpatient diagnoses following the transition to the ICD-10 coding system. This upward trend likely reflects improved recognition and coding rather than a true surge in incidence. The increasing trends in these venous conditions underscore the need for heightened clinical awareness and targeted interventions to manage these conditions effectively. As the National Inpatient Sample captures only hospitalized patients, these findings should be interpreted as inpatient diagnostic trends and do not fully represent the overall prevalence or outpatient management of PCS.

**Article Highlights**

• **Type of Research:** Multicenter retrospective cohort study using the National Inpatient Sample
• **Key Findings:** Pelvic Congestion Syndrome (PCS) diagnoses rose sharply after the International Classification of Diseases, 10th Revision transition in 2015, suggesting improved recognition and coding rather than a true increase in disease incidence.
• **Take Home Message:** Despite affecting millions of women, PCS remains underdiagnosed and under-recognized. This study shows that although diagnostic rates have improved—especially after implementation of the International Classification of Diseases, 10th Revision—systemic disparities and low clinical prioritization persist. Greater awareness, education, and standardized guidelines may close the gap in PCS recognition and care.

Chronic pelvic pain (CPP) accounts for nearly 10% of all gynecological complaints among women of reproductive age.^1^ Although this symptom can be multifactorial in origin, clinical workups often focus on ruling out well-known accepted conditions such as endometriosis, uterine leiomyomas, or malignancy. In contrast, pelvic congestion syndrome (PCS) —a condition characterized by CPP persisting for over 6 months that is exacerbated by sexual activity, menstruation, or prolonged standing—remains under evaluated and misunderstood etiology.^2^

PCS is associated with pelvic venous insufficiency, particularly ovarian and internal iliac varicosities. It predominantly affects multiparous women of childbearing age and is believed to have hormonal influences, as symptoms often abate after menopause.^3^ Diagnostic imaging such as duplex ultrasound study or venography can reveal hallmark findings of venous reflux and pooling, which contribute to pelvic nerve compression and visceral pain commonly reported by affected patients.^4^

Despite increasing evidence supporting effective treatment of PCS,^5^ such as coil embolization and sclerotherapy, many specialists believe that the pathology continues to be underdiagnosed and undertreated. Moreover, due in part to its nonspecific symptoms, PCS has historically suffered from stigma and limited clinical prioritization that an objective observer could not help but attribute to gender prejudice.^6^ The Society of Vascular Surgeons (SVS) now recommends treating PCS and pelvic varices with endovascular interventions, which have shown promising results in improving quality of life in multiple studies.^7^, ^8^, ^9^, ^10^ But there is yet to be comprehensive guidelines, or high-grade evidence recommendations, on management and intervention on these patients that most likely is attributed to the paucity of research done on this pathology.

The transitions from International Classification of Diseases, Ninth Revision (ICD-9) to International Classification of Diseases, 10th Revision (ICD-10) in 2015 introduced more granular diagnostic coding and have facilitated more accurate capture of previously undercoded conditions, such as PCS. This, along with growing awareness and advances in therapeutic options, provides a unique opportunity to reevaluate national trends in PCS diagnoses and treatment patterns. The primary aim of this study is to describe the evolving epidemiology of PCS using national inpatient data, with a focus on identifying under-recognized patterns, disparities in diagnosis and care, and how PCS compares with other venous pathologies such as chronic venous insufficiency (CVI) and varicose veins (VV). Through this, we aim to highlight the possible continued stigma surrounding PCS and in turn, advocate for greater clinical recognition.

## Methods

### Data source

A retrospective cohort analysis of inpatient admissions of patients with PCS, VV, and CVI using the Nationwide Inpatient Sample (NIS) was performed. The data were obtained from the NIS from 2004 through 2021, which represents nearly 95% of all inpatient admissions in 48 of 50 US states.^11^, ^12^, ^13^ The Cooper University Hospital Institutional Review Board approved the present study and waived the need for written informed consent owing to the retrospective and deidentified nature of the NIS database.

### Study population

All patients within the NIS with a diagnosis code in any position for pelvic varices, PCS, VV, and CVI were identified. Vulvar varices were not analyzed separately because of inconsistent coding granularity across the ICD-9 to ICD-10 transition. We acknowledge that lower-extremity VV/CVI and pelvic venous disease are anatomically and clinically distinct; therefore, comparisons with VV and CVI should be interpreted only as contextual comparisons of national venous-disease coding trends, rather than as direct disease equivalents. For years 2004 to 2015 through quarter three, patients were identified using ICD-9-CM codes, whereas for quarter four of 2015 to 2019, the patients were identified using ICD-10-CM codes. Then, using US Census data, annual prevalence of PCS was calculated per 100,000 population each year.^14^ Patients who underwent procedures while inpatient were then identified, including hypogastric vein procedure, abdominal vein procedures, leg vein procedures, and sclerosant injections using ICD-9 and ICD-10 procedural codes (ICD codes used are presented in Supplementary Table I, online only). Of note, due to the nature of ICD coding, there was no further description of the specific procedures included with each code. For example, “leg vein procedure” includes phlebectomy and radiofrequency ablation, among others, but does not go into specific details regarding the number of each specific procedure. To ensure consistent data capture through the period of time when the United States converted from ICD-9-CM to ICD-10-CM, extensive attempts were made to make accurately equivalent conversions for pathologies and procedures from ICD-9-CM to ICD-10-CM^15^, ^16^, ^17^, ^18^ (Supplementary Table II, online only).

For all patients, relevant demographic data as well as important comorbidities using Elixhauser covariates^19^ (2004-2012) and associated ICD codes (2012-2019) were collected. Hospital data were collected including hospital setting, whether the hospital was classified as a rural, urban nonteaching, urban teaching hospital. Similarly, the US region of the hospital, either Northeast, Midwest, South, or West, was collected.

The primary outcomes were the trends in diagnoses of each of the venous pathologies, mainly PCS. Secondary outcomes included inpatient interventions performed on the venous pathologies as well as where the patient was regionally and within what hospital setting. Importantly, there are no ICD-9 or ICD-10 procedure codes specifically for ovarian vein embolization.

### Statistical analysis

Categorical variables were represented as frequencies and percentages, whereas continuous variables were presented as mean and standard deviation if they had a normal Gaussian distribution. Categorical variables were compared using the Pearson χ^2^ test, whereas continuous variables were compared using the Wald test to assess for differences in the mean. We used the Cochrane-Armitage *P* value trend test for intervention method proportions and mortality over the years. All statistical analyses were performed using Stata, version 18.0 (StataCorp LLC).

## Results

### Trends of pelvic varices, PCS, VV, and CVI

**Fig 1:**
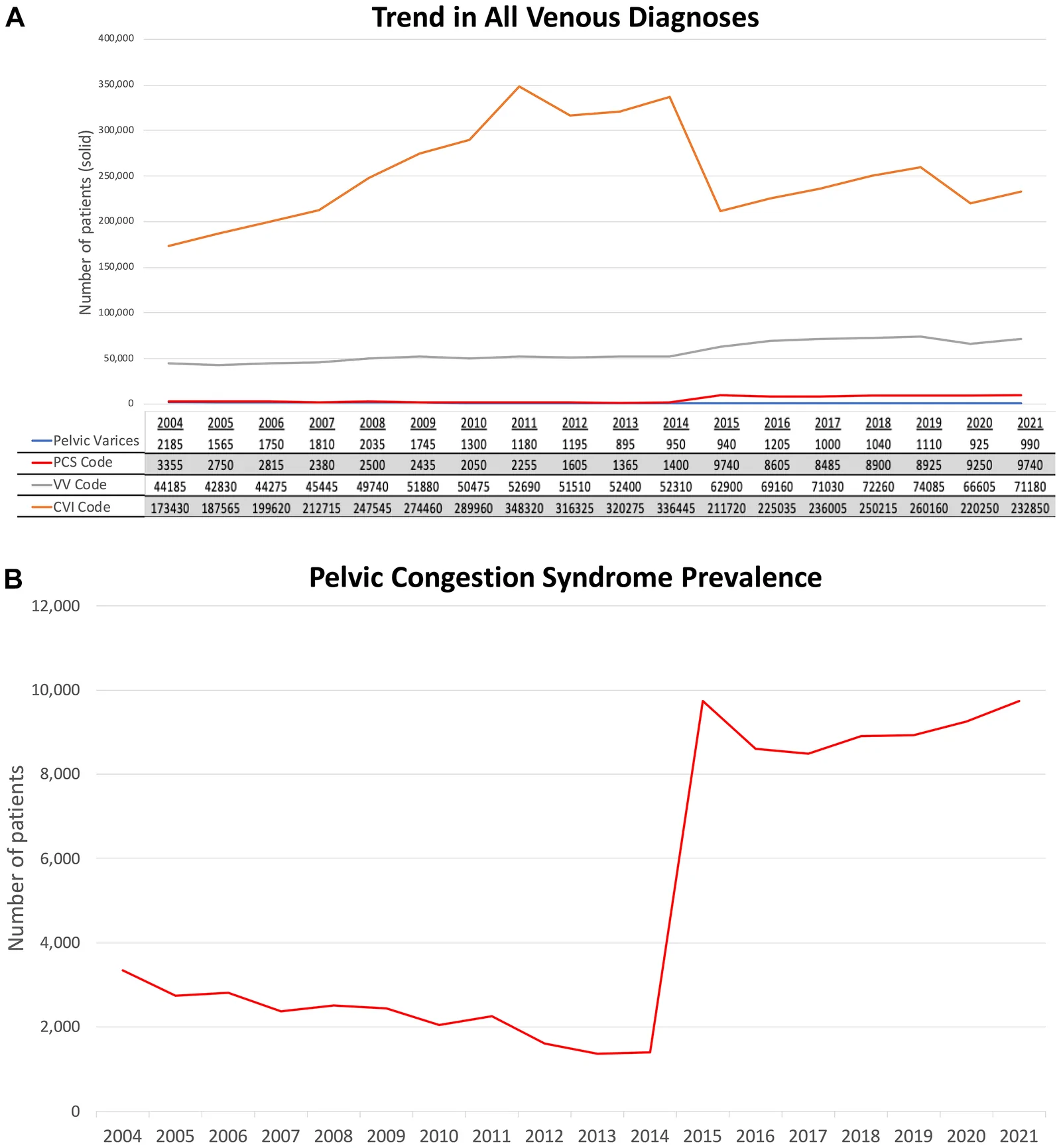
Trends in prevalence of all venous pathologies from 2004 to 2021 **(A)** and prevalence per 100,000 of pelvic congestion syndrome (*PCS*) alone **(B)**. *CVI*, Chronic venous insufficiency; *VV*, varicose veins.

Over the study period between 2004 and 2021, a total of 23,820 patients with pelvic varices, 88,555 patients with PCS, 1,024,960 patients with VV, and 4,542,895 patients with CVI were identified. From 2004 to 2021, there was an increase in the number of patients diagnosed with CVI, VV, and pelvic venous disorders, as depicted in Fig 1, *A*. CVI remained the most frequently coded diagnosis, rising from 173,430 cases in 2004 to a peak of 348,320 in 2011 before stabilizing between 220,000 and 250,000 annually in the following years (*P*-trend < .001). VV diagnoses followed a similar trend, increasing steadily from 44,185 in 2004 to a peak of 74,085 in 2019.

In contrast, diagnoses associated with pelvic venous pathologies, including pelvic varices and PCS, remained substantially lower throughout the study period. Patients with PCS remained below 10,000 per year, with a substantial increase observed after the ICD coding conversion in 2015 (2004-2021: 3355-9740; *P*-trend < .001). With the increase in PCS diagnoses, we saw a concordant decrease in diagnoses of pelvic varices over the study period, from 2185 in 2004 to 990 in 2021 (*P*-trend < .001). The prevalence of PCS per 100,000 US population increased over the study period, from 1.14/100,000 in 2004 to 2.93/100,000 in 2021 (*P*-trend < .001; Fig 1, *B*). Again, a stark increase was noted with the conversion from ICD-9 to ICD-10 in 2015, nearly sixfold, after decreasing from 2004 to 2014 (1.14/100,000 to 0.44/100,000).

### Demographic and comorbidities within patients with venous pathologies

Patients with PCS were significantly younger, with a mean age of 47.3 years, compared with 67.9 years among those with VV and 70.5 years among those with CVI. All patients with PCS were female, whereas females comprised 53.5% of patients with VV and 47.8% of those with CVI. The racial distribution within the PCS group mirrored the overall racial distribution across the United States, with 54.0% of patients being White, 15.2% being Black, 13.9% being Hispanic, and 3.1% being Asian.

With PCS patients being much younger, they were also a much healthier cohort exhibiting less comorbid conditions. Only 33.7% of patients with PCS had hypertension, compared with 73.9% in the VV group and 81.7% in the CVI group. Similar trends were observed across other comorbidities, including chronic kidney disease, diabetes mellitus, congestive heart failure, and peripheral vascular disease (Table).

**Table:** Demographics and comorbidities of pelvic congestion syndrome (*PCS*), chronic venous insufficiency (*CVI*), and varicose veins (*VV*)

Variables	PCS		VV		CVI	
No. of patients	88,555		1,024,960		4,542,895	
Age, years, mean (SD)	47.27	0.21	67.9	0.142	70.46	0.136
	n	f	n	f	n	f
Sex						
Male	0	0	476,605	46.5	2,371,390	52.2
Female	88,555	100.0	548,355	53.5	2,171,505	47.8
Race						
White	47,820	54.0	703,125	68.6	3,334,485	73.4
Black	13,460	15.2	107,620	10.5	467,920	10.3
Hispanic	12,310	13.9	86,095	8.4	245,315	5.4
Asian	2745	3.1	12,300	1.2	45,430	1.0
Other	12,130	13.7	115,820	11.3	449,745	9.9
Comorbidities						
HTN	29,845	33.7	757,445	73.9	3,711,545	81.7
CKD	6730	7.6	260,340	25.4	1,608,185	35.4
DM	11,955	13.5	365,910	35.7	2,053,390	45.2
CHF	5755	6.5	332,085	32.4	2,012,500	44.3
PVD	1860	2.1	145,545	14.2	617,835	13.6

*CHF*, Congestive heart failure; *CKD*, chronic kidney disease; *DM*, diabetes mellitus; *HTN*, hypertension; *PVD*, peripheral vascular disease; *SD*, standard deviation.

Categorical data are shown as number (%).

### Trends of inpatient venous procedures performed

**Fig 2:**
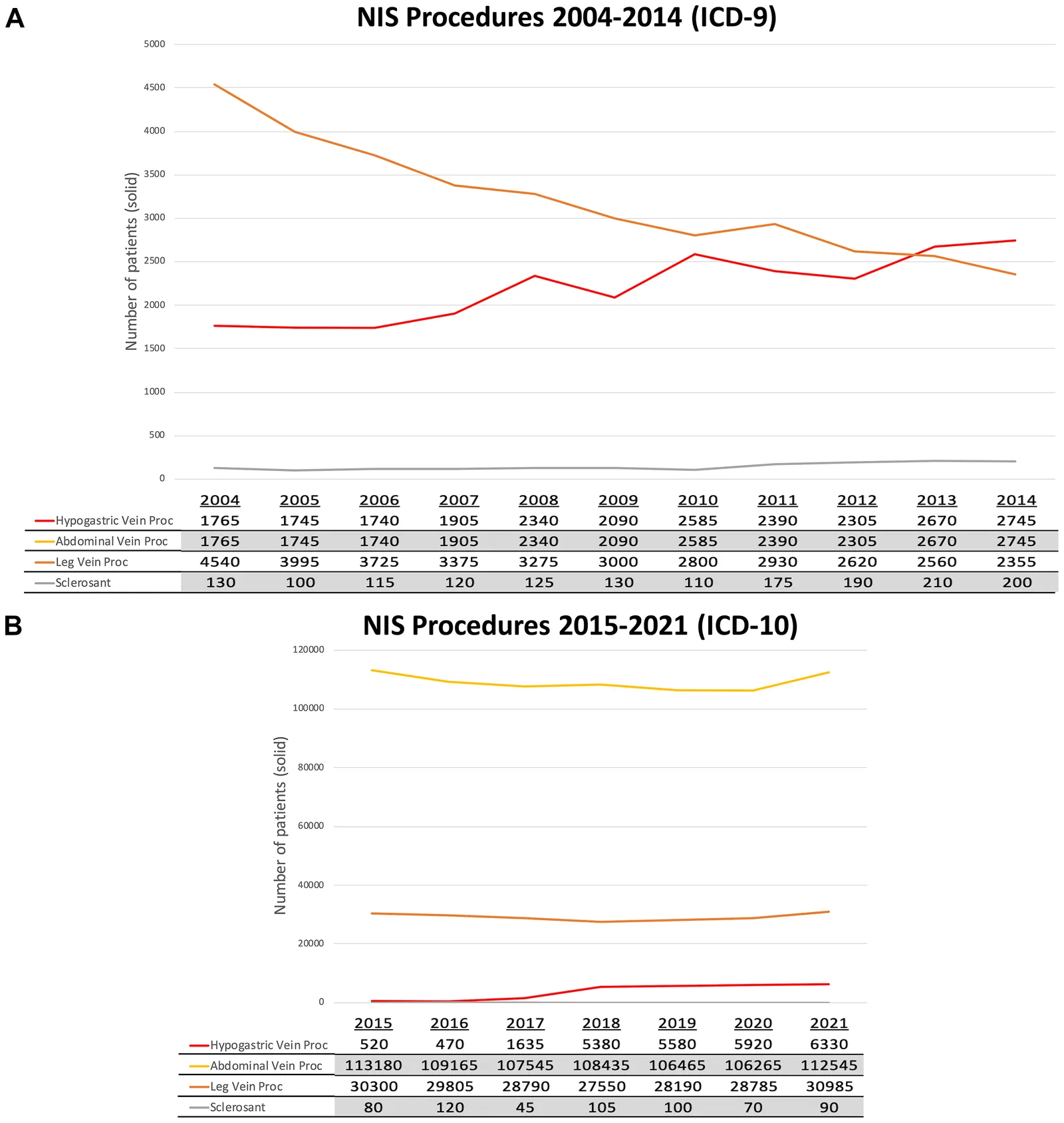
Trends of inpatient procedures performed for venous pathologies: International Classification of Diseases, Ninth Revision (*ICD-9*) from 2004 to 2014 **(A)** and International Classification of Diseases, 10th Revision (*ICD-10*) from 2015 to 2021 **(B)**. *NIS*, National Inpatient Sample.

Across the ICD-9 coding system, from 2004 to 2014, leg vein procedures were much more common than hypogastric or abdominal vein procedures, which shared an ICD-9 procedural code. Inpatient leg vein procedures decreased by half, from 4540 in 2004 to 2355 in 2014. In contrast, inpatient hypogastric and abdominal vein procedures increased from 1765 in 2004 to 2745 in 2014 (Fig 2).

With the conversion to ICD-10, a stark increase in abdominal and leg vein procedures was observed. Hypogastric vein procedures saw a stark increase, with the introduction of a unique code, from 520 in 2015 to 6330 in 2021. Leg vein procedures remained stable, with 30,300 leg vein procedures being performed in 2015 and 30,985 in 2021.

### Trends of venous pathologies across US regions

**Fig 3:**
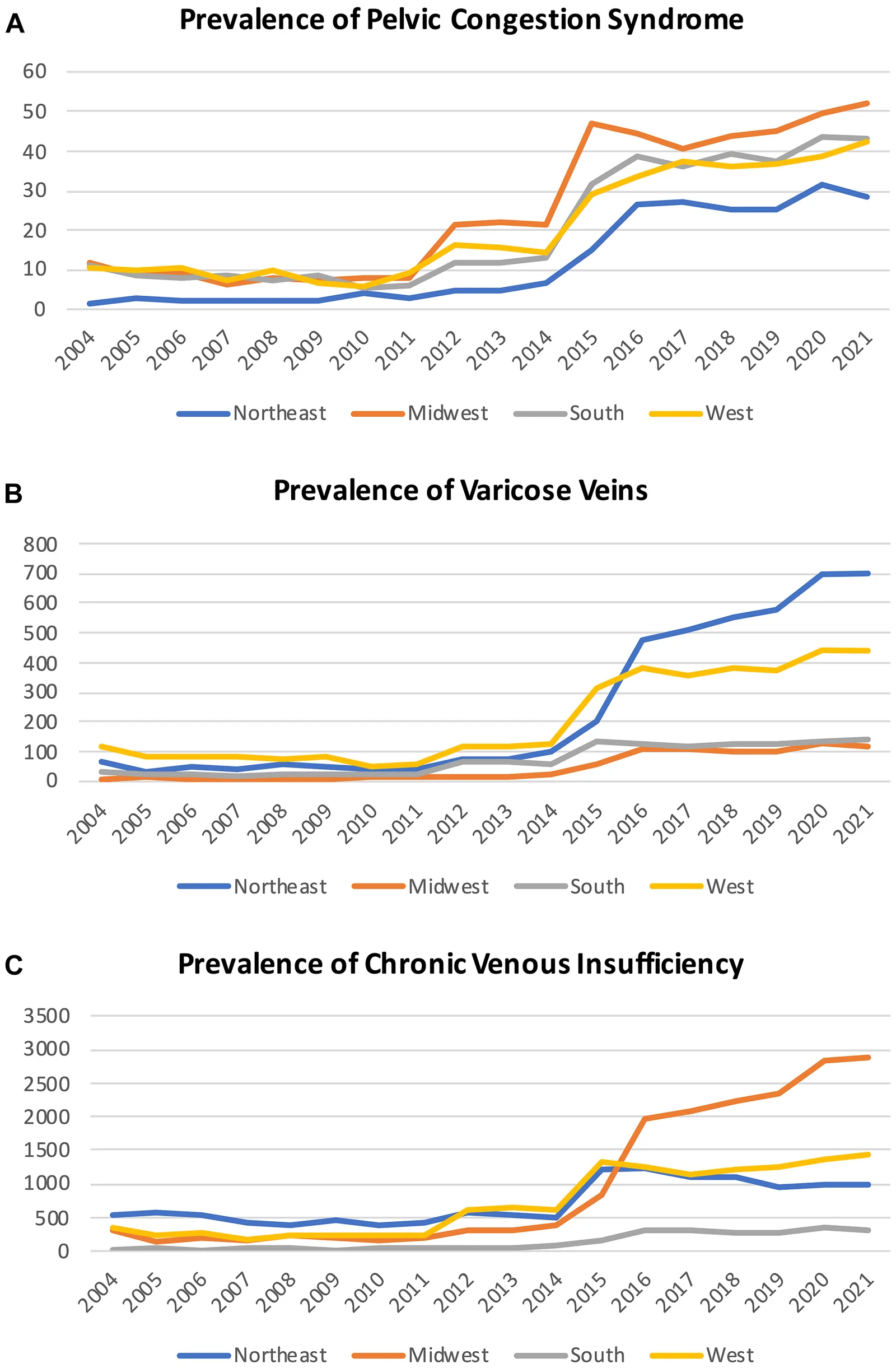
Trends of all venous pathologies stratified by US regions (Northeast, Midwest, South, and West): pelvic congestion syndrome **(A)**, varicose veins **(B)**, and chronic venous insufficiency **(C)**.

The prevalence of PCS increased substantially across all US regions from 2004 to 2021 (all *P*-trend < .001; Fig 3, *A*), though the magnitude varied between regions. Hospitals in the Northeast consistently had the lowest number of patients with PCS, whereas hospitals in the Midwest consistently had the highest number of patients with PCS. In 2004, hospitals in the Northeast saw 1.4 patients with PCS per 100,000 hospitalized patients, whereas hospitals in the Midwest, South, and West all saw about 10.0 patients with PCS per 100,000 hospitalized patients. However, hospitals in the Northeast experienced the greatest overall rise in patients with PCS over the study period (2004-2021: 1.4-28.1/100,000). In 2021, Midwest hospitals saw the greatest rate of patients with PCS (52.1/100,000), nearly double the rate of their Northeast counterparts. It should be noted that marked rises in PCS diagnoses occurred between 2011 and 2016, even before the introduction of ICD-10, which was not seen in the other venous pathologies.

VV was overall more prevalent, 100-700/100,000 hospitalized patients in 2021, with much sharper regional disparities with opposite trends of PCS. Hospitals in the Midwest consistently had the lowest number of patients with VV (6.6-115.7/100,000), with hospitals in the South having a similar experience. From 2004 to 2013, VV diagnosis rates were relatively stable and modest across all regions; however, beginning in 2014 the Northeast and the West experienced a striking rise in VV prevalence, reaching 699.5 and 439.7 per 100,000 hospitalized patients in 2021, respectively (both *P*-trend < .001; Fig 3, *B*).

CVI was overall even more prevalent, with 300-3000/100,000 hospitalized patients in 2021. Similar to PCS, hospitals in the Midwest saw the greatest number of patients with CVI (Fig 3, *C*). After maintaining relatively low and stable rates (∼150-300 per 100,000 hospitalized patients) between 2004 and 2013, there was a steep rise beginning in 2014. By 2015, the prevalence had more than doubled to 823/100,000 and continued to increase, reaching 2878.9/100,000 hospitalized patients in 2021. In 2021, hospitals in the Midwest saw patients with CVI at least double that of any other region in the United States (per 100,000: Northeast 984.0, South 314.0, West 1433.9). Although hospitals in the Northeast and the West saw more modest increases, conversely hospitals in the South saw a largely stable rate, never reaching above 314.0/100,000.

### Trends of venous pathologies at different hospital settings

**Fig 4:**
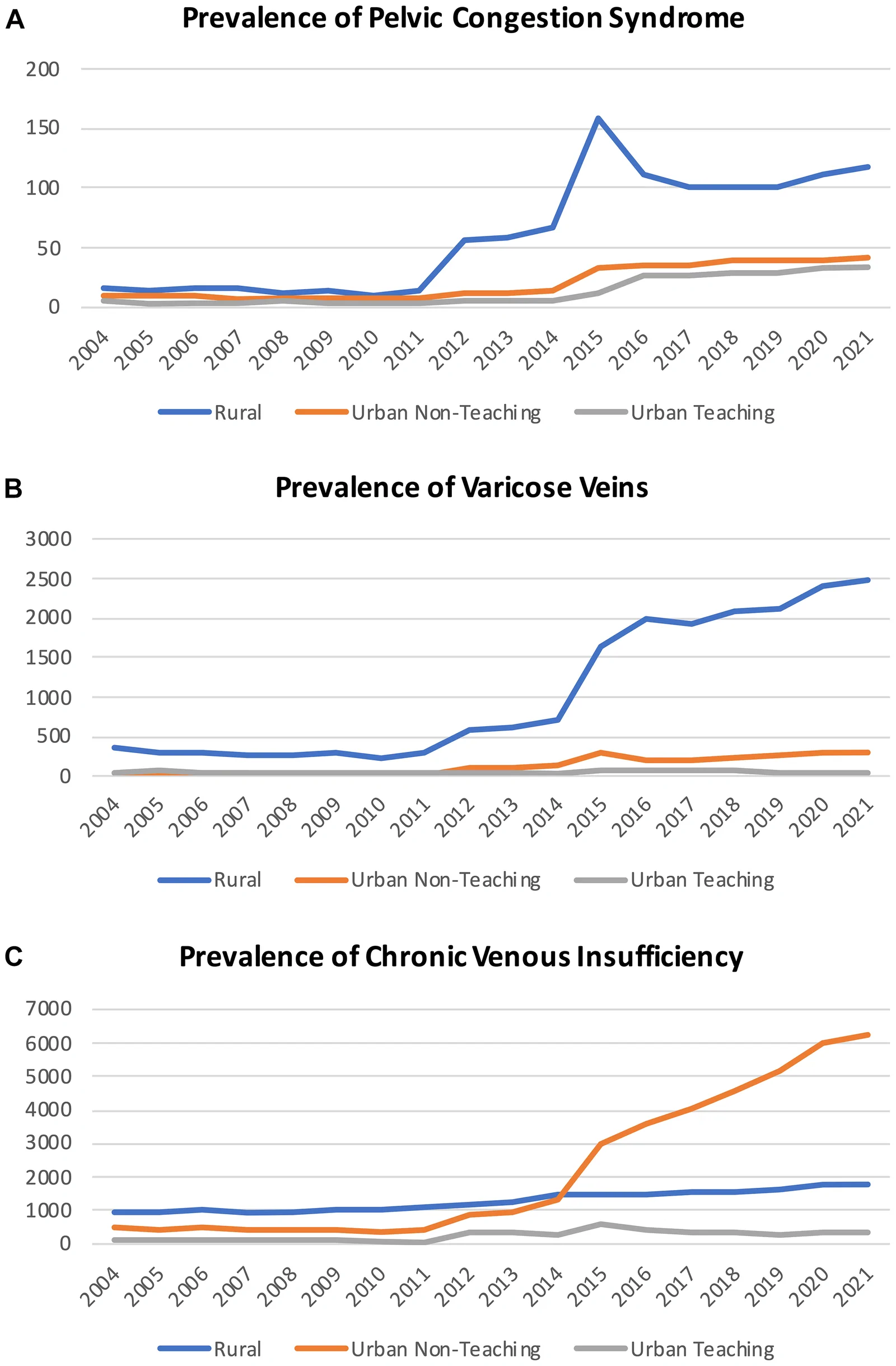
Trends of all venous pathologies stratified by hospital setting (rural, urban nonteaching, urban teaching): pelvic congestion syndrome **(A)**, varicose veins **(B)**, and chronic venous insufficiency **(C)**.

PCS increased across all hospital settings (all *P*-trend < .001), with markedly different trends between rural and urban hospitals. Rural hospitals consistently saw the highest rate of patients with PCS (Fig 4, *A*). From 2004 to 2011, the rate of PCS at rural hospital was stable between 10 and 17 cases per 100,000 hospitalized patients, before surging in 2012 and continually increasing through 2021 to reach 117.7 cases per 100,000 hospitalized patients. Urban-teaching and nonteaching hospitals had more stable rates except for an increase that occurred in conjunction with the adoption of ICD-10 in 2015, with urban-teaching hospitals consistently having the lowest rates of patients with PCS (2004-2021: 6.0-33.8/100,000 hospitalized patients).

VV diagnoses increased across all hospital settings, though greatly differed in magnitude depending on what the hospital setting was. There were strikingly higher rates of VV at rural hospitals compared with both urban-nonteaching and urban-teaching hospitals (Fig 4, *B*). Rural hospitals consistently had the highest rates and the fastest growth in VV diagnoses, rising from 353.9 cases in 2004 to 2748.2 cases in 2021, per 100,000 hospitalized patients. Urban-teaching hospitals again showed relatively stable rates throughout the study period, increasing from 29.2 cases to 70.3 cases per 100,000 patients from 2004 to 2021.

CVI had the greatest difference in trends between hospital settings of the venous pathologies. Urban-teaching hospitals, similar to VV and PCS, saw consistently the lowest rate of CVI compared with other hospital settings (2004-2021: 121.8-337.0 cases per 100,000 hospitalized patients; Fig 4, *C*). Conversely, urban-nonteaching hospitals rose to be the hospital setting that most frequently saw patients with CVI, with 6241.8 cases per 100,000 hospitalized patients in 2021.

## Discussion

PCS can be the underlying cause of otherwise unexplained CPP^20^ affecting an estimated 6.5 million reproductive-aged women in the United States.^21^ PCS is a significant contributor to pelvic pain in women, causing as much as 30% of CPP.^22^ Diagnosis may be missed or delayed due to vague symptoms that can mimic those of other conditions, making accurate diagnosis particularly challenging. Limited awareness and/or acceptance of the syndrome may also contribute. We present a rising trend in PCS diagnoses across the United States since 2015 that is most likely not a representation of a true epidemiologic increase in incidence, but rather a demonstration of increased awareness and acceptance of the pathology as a true cause of patients' pelvic pain.

CPP is highly prevalent, comparable to the global prevalence of asthma^23^ and the 1-month prevalence of low back pain.^20^ For women of reproductive age, the prevalence is between 14% and 24%.^24^^,^^25^ CPP has a wide and multisystem differential diagnosis, with possible etiologies from the gastrointestinal system, the renal system, or the genitourinary systems. Commonly, these patients with irritable bowel syndrome, interstitial cystitis, endometriosis, or PCS may all describe unilateral dull ache or heaviness in the pelvis for 3 to 6 months. However, a distinguishing feature of PCS is the presence of postcoital pain and adnexal tenderness on physical examination. A meta-analysis by O’Brien et al^26^ reported that the combination of these two findings—ovarian point tenderness and postcoital pain—demonstrated a sensitivity of 94% and a specificity of 77% for PCS diagnosis. These clinical signs are critical, especially given the nonspecific nature of many other symptoms associated with PCS, including dyspareunia, bladder irritability, heaviness in the pelvis, and exacerbation of pain with standing or during the premenstrual period. Furthermore, physical examination may reveal vulvar or lower-extremity varicosities, although these findings are not required for diagnosis.

The missed diagnosis of PCS certainly can be attributed to the nonspecific symptoms, but may also be due to the lack of awareness and/or acceptance of PCS as a true etiology of CPP by physicians who would be the referral pathways to interventionalists who could evaluate, diagnose, and subsequently selectively treat symptomatic ovarian varicosities. The American College of Gynecologists practice bulletin on CPP, gynecologists societal published guideline, mention a large list of differential diagnoses that may be the cause of women's pelvic discomfort, but it lacks the mention of PCS. However, they note that “although venous congestion appears to be associated with CPP, evidence is insufficient to conclude that there is a cause-and-effect relationship.^27^” The American Academy of Family Physicians article on CPP does mention PCS as part of their comprehensive differential diagnoses list,^28^ but they do not list as “highly associated with CPP” as they do other etiologies that can be equally prevalent, nor do they mention the necessitation to refer the patient to a vascular surgeon.

Additional factors to the low diagnosis rate of PCS may be due to inherent biases within medicine as a whole. We present the most recent prevalence among the female population to be 9205, whereas the estimated prevalence should be in the millions per various population studies.^22^^,^^29^^,^^30^ Extensive research underscores systemic gender disparities in health care, revealing that women often face delayed diagnoses, inadequate pain management, and dismissal of their symptoms. Women are disproportionately affected by chronic pain conditions. However, they often receive less effective pain relief, are prescribed more antidepressants, and are more frequently referred to psychiatric services, reflecting potential biases in pain assessment and management.^31^, ^32^, ^33^ A study analyzing emergency department datasets found that female patients are less likely to be prescribed pain-relief medications compared with males across many chief complaints.^34^

In addition, studies have shown that women with CPP demonstrate increased pain sensitivity at nonpelvic sites compared with healthy controls, suggesting central pain amplification.^35^ This heightened sensitivity is often overlooked, leading to misdiagnoses or attributing symptoms to psychological causes.^36^ The underrepresentation of women in clinical research contributes to gaps in understanding and treating CPP. Historically, medical studies have predominantly involved male subjects, leading to a lack of data on how treatments affect women differently. This male-centric focus has resulted in diagnostic tools and treatment protocols that may not adequately address women's health needs.^37^

Although PCS is still likely underdiagnosed, our study does show modest improvements in the acceptance of PCS. Spanning the conversion of ICD-9 to ICD-10, the observed rise in diagnosis of PCS merits particular attention. In 2014 using the ICD-9 coding system there were 1400 inpatients with a diagnosis of PCS, then in 2015 using the ICD-10 coding system this number increased to 9740 patients. The observed rise in prevalence of PCS following the transition to ICD-10 likely does not reflect a true epidemiological increase, but rather increased awareness and diagnosis of the traditionally stigmatized disease. This observation can also be, in part, due to the improved disease classification afforded by the more detailed coding and structure of ICD-10, which most likely accounts for the rise in prevalence of VV and CVI over the ICD transition period.

Although there was not an observed increase in PCS diagnoses from 2015 to 2021, there is hope for improved diagnosis rates. In 2021, the Symptoms-Varices-Pathophysiology classification was introduced and will further facilitate clinical communication and the development of patient-reported outcome measures.^38^ The use of historical nomenclature for pelvic venous disorders fails to recognize the complex and interrelated pelvic venous circulation, contributes to misdiagnosis and poor treatment outcomes, and hinders clinical research.^38^

The observed decline in the prevalence of pelvic varices over the study period could be due to a few factors. Although this trend may suggest a true epidemiological reduction, it is more likely reflective of evolving diagnostic and documentation practices such as diagnostic overshadowing, wherein pelvic varices—previously coded as distinct entities—are now being coded under broader or more clinically emphasized diagnoses such as PCS or CVI. In addition, advances in imaging technology and techniques may contribute to this trend. For instance, cross-sectional imaging such as magnetic resonance venography and transvaginal Doppler ultrasound examination now allow for better visualization of pelvic venous congestion, potentially leading to a diagnostic label of PCS rather than isolated pelvic varices. Similarly, the rise in incidence of CVI is potentially due to more accurate diagnoses made on noninvasive testing and increased availability of such testing.

Despite the rising number of diagnoses for PCS, VV, and CVI, the incidence of inpatient procedures related to abdominal venous disorders and leg venous occlusions remained largely unchanged throughout the study period. This finding likely reflects, at least in part, a national shift of venous interventions from inpatient to outpatient and ambulatory settings, driven by evolving reimbursement structures, advances in minimally invasive techniques, and increasing use of office-based endovascular procedures. This also, potentially, reflects a trend toward conservative management, particularly in early or moderate cases, favoring medical therapy, lifestyle modifications, or outpatient-based interventions over inpatient surgical or endovascular procedures. This finding may also highlight health care access disparities, where certain populations—especially underserved or low-income patients—may face barriers in obtaining specialized venous care. Procedure utilization may also be constrained by the availability of interventional radiology or vascular surgery services in some inpatient settings, or by lack of clinical consensus on intervention thresholds for PCS. For the treatment of PCS and pelvic varices, the SVS suggests the use of coil embolization, vascular plugs, or transcatheter sclerotherapy. If less invasive treatments are unavailable or have proven unsuccessful, surgical ligation and excision of the ovarian veins to address reflux are suggested.^4^^,^^39^

For the treatment of PCS and pelvic varices, the SVS suggests the use of coil embolization, vascular plugs, or transcatheter sclerotherapy. If less invasive treatments are unavailable or have proven unsuccessful, surgical ligation and excision of the ovarian veins to address reflux are suggested. More recent procedural outcomes studies have further emphasized that pelvic venous disorder is not a uniform reflux-only pathology and that treatment may require addressing associated venous obstruction. Lakhanpal et al^40^ reported clinical improvement after iliac vein stenting alone in patients with symptomatic pelvic venous insufficiency secondary to combined iliac vein stenosis and ovarian vein reflux. More recently, Trzesniowski et al^41^ provided long-term follow-up data supporting this approach in selected patients with combined iliac vein stenosis and ovarian vein reflux treated with iliac vein stenting alone. These findings highlight the importance of individualized anatomic and hemodynamic assessment when considering intervention for pelvic venous disorder.

The geographic and institutional differences in PCS prevalence further underscore these systemic gaps. Notably, hospitals in the Midwest reported the highest PCS diagnosis rates, whereas those in the Northeast reported the lowest. Interestingly, the Northeast experienced the steepest rise over time, potentially due to growing adoption of advanced imaging and multidisciplinary pelvic pain clinics in academic centers. Rural hospitals also consistently documented higher PCS and VV prevalence compared with their urban-teaching counterparts. These findings may reflect differences in diagnostic focus, with rural hospitals providing greater attention to venous disease. Alternatively, higher PCS prevalence in the Midwest and the South may correlate with known regional risk factors such as obesity, multiparity, and limited access to specialty care.

Our study offers one of the most comprehensive assessments of PCS and related venous pathologies in the United States, leveraging data from the NIS across an 18-year span. Although this analysis is based on an inpatient database, the diagnostic coding used reflects patients' entire documented medical histories rather than conditions limited to the admission event. This significantly strengthens the epidemiological relevance of our findings. Patients admitted for unrelated diagnoses (eg, myocardial infarction) still contributed to PCS prevalence figures if the condition was known and coded. This suggests our prevalence estimates more accurately reflect national disease burden compared with strictly encounter-limited datasets.

Our study has several limitations. The use of administrative data from the NIS may be subject to coding inaccuracies and inconsistencies over time. In addition, inpatient data may underrepresent the total burden of PCS and VV, which are often managed in outpatient settings. Accordingly, the procedural volumes reported in this study should be interpreted as inpatient procedural trends only and not as representative of total national venous intervention volume. In this context, our findings reinforce the necessity of standardizing diagnostic criteria for PCS and related venous disorders to further improve coding consistency and ensure reliable epidemiological surveillance.

The study highlights the rising number of diagnoses of PCS, VV, and CVI over the study period, particularly after the transition to the ICD-10 coding system. Accurately identifying patients whose symptoms are genuinely attributable to PCS is essential for providing tailored and effective therapeutic interventions.

## Author Contributions

Conception and design: PC

Analysis and interpretation: AG, PC, CD, DJ, JL, LH, KM

Data collection: PC, AP

Writing the article: AG, PC, CD

Critical revision of the article: AG, PC, AP, DJ, JL, LH, KM

Final approval of the article: AG, PC, CD, AP, DJ, JL, LH, KM

Statistical analysis: Not applicable

Obtained funding: Not applicable

Overall responsibility: PC

AG an PDC contributed equally to this article and share co-first authorship.

## Funding

None.

## Disclosures

None.

## Appendix (online only)

Supplementary Table I and II (online only)
