# Supplementary material for: The national acceptance of pelvic congestion syndrome among other common venous pathologies
Source: J Vasc Surg Venous Lymphat Disord. 2026 Aug 10;14(6):102596. doi: 10.1016/j.jvsv.2026.102596 (PMC13560327; doi:10.1016/j.jvsv.2026.102596)
Supplement: Supplementary file 1 [file mmc1.docx]

Supplemental Table1: ICD-9-CM and ICD-10-CM Codes Used

| ICD9 DX | PCS | 6255x = Pelvic Congestions Syndrome |
| --- | --- | --- |
|  | CVI | 45981 = Venous (peripheral) insufficiency, unspecified |
|  |  | 4593x = Chronic venous hypertension (idiopathic) |
|  | VV | 454xx = Varicose veins of lower extremities |
|  | Vulvar Varices | 4566x = Vulvar Varices |
| ICD10 DX | PCS | N9489 = Other specified conditions associated with female genital organs and menstrual cycle |
|  | CVI | I862 = Pelvic Varices |
|  |  | I872 = Venous insufficiency (chronic) (peripheral) |
|  | VV | I83x = Varicose veins of lower extremities |
|  | Vulvar Varices | I863 = Vulvar Varices |

Supplemental Table 2:Checklist for studies published using National Inpatient Sample survey data

| Section A: Research Design | |
| --- | --- |
| 1. Does the study consider that it can only detect diseases conditions, procedures, and  diagnostic tests in hospital settings? | **Yes** |
| 2. Does the study acknowledge that it includes encounters, not individual patients? | **Yes;** we have used “hospitalizations” in place of “patients” wherever it is applicable |
| Section B: Data Interpretation | |
| 3. Does the study attempt to identify disease conditions or procedures of interest using  administrative codes or their combinations that have been previously validated? | **Yes**; we have identified all the disease conditions or procedures using the previously validated and/or published administrative codes |
| 4. Does the study limit its assessment to only in-hospital outcomes, rather than those  occurring after discharge? | **Yes** |
| 5. Does the study distinguish complications from comorbidities or clearly not where it cannot? | **Yes** |
| Section C: Data Analysis | |
| 6. Does the study clearly account for the survey design of the NIS and its components—  clustering, stratification, and weighting? | **Yes;** All analyses were conducted accounting for survey nature (SURVEYMEANS,  SURVEYLOGISTIC, and SURVEYFREQ), clustering (HOSP_NIS), weighting (DISCWT),  and stratification (NIS_STRATUM) of the NIS. |
| 7. Does the study adequately address changes in data structure over time (for trend  analyses)? | **Yes;** this is addressed this in the methods section |
